# Fatigue-Free Force-Velocity and Power-Velocity Profiles for Elite Track Sprint Cyclists: The Influence of Duration, Gear Ratio and Pedalling Rates

**DOI:** 10.3390/sports10090130

**Published:** 2022-08-31

**Authors:** Anna Katharina Dunst, Clemens Hesse, Olaf Ueberschär, Hans-Christer Holmberg

**Affiliations:** 1 Department of Endurance Sports, Institute for Applied Training Science, 04109 Leipzig, Germany; 2 German Cycling Federation, 60528 Frankfurt, Germany; 3 Department of Biomechanics, Institute for Applied Training Science, 04109 Leipzig, Germany; 4 Department of Engineering and Industrial Design, Magdeburg-Stendal University of Applied Sciences, 39114 Magdeburg, Germany; 5 Department of Health Sciences, Luleå University of Technology, 971 87 Luleå, Sweden; 6 Department of Physiology and Pharmacology, Biomedicum C5, Karolinska Institutet, 171 77 Stockholm, Sweden

**Keywords:** F/v-profile, validity, track cycling, fatigue-free performance, performance diagnostics

## Abstract

Background: Maximal force-velocity (F/v) profiles for track cyclists are commonly derived from ergometer sprints using an isovelocity or isoinertial approach. Previously, an attempt was made to derive maximal F/v profiles from a single maximal 65-m sprint on the cycling track. Hypothesising that this approach may not accurately reflect the fatigue-free F/v profile, we propose an alternative procedure and compare it to the previous method. Moreover, we test for the impact of gear ratio on diagnostic results. Methods: Twelve elite track cyclists completed a high-cadence low-resistance pedalling test on a freestanding roller (motoric test) and two series of three maximal 65-m sprints on a cycling track with different gear ratios. F/v profiles were calculated based on the measured crank force and cadence either during the first 6–7 revolutions (≤6 s) on the track (model I) or were derived from the first 3–4 revolutions (≤3 s) on the track combined with 1 or 2 fatigue-free cycles at cadences above 160 rpm from the motoric test (model II). Results: Although both models exhibit high-to-excellent linearity between force and velocity, the extrapolated isometric force was higher (1507.51 ± 257.60 N and 1384.35 ± 276.84 N; *p* < 0.002; d = 2.555) and the slope steeper (−6.78 ± 1.17 and −5.24 ± 1.11; *p* < 0.003, d = −2.401) with model I. An ICC of 1.00 indicates excellent model consistency when comparing the F/v profiles (model II) derived from the different geared sprints. Conclusions: Assuring fatigue-free measurements and including high-cadence data points in the calculations provide valid maximal F/v and P/v profiles from a single acceleration-sprint independent of gear ratio.

## 1. Introduction

A decisive physiological determinant of sprint performance in track cycling is the ability to produce fatigue-free muscular power, which can be described with maximal force-velocity (F/v) and power-velocity (P/v) profiles. Although A. V. Hill [1] modelled the velocity of the shortening of contractile elements as an inverse hyperbola, the force-velocity profiles of large muscle groups acting upon more than a single joint may be adequately approximated by linear relations [2]. Especially in the case of multi-joint movements with resistance, such as those involved in jumping, running, cycling, lifting and throwing, almost linear relations between generated force and body segment velocity have widely been found [3].

In sprint cycling, a strong linear relation of mean pedal force (F) and pedalling rate (PR) has been reported in numerous investigations (e.g., [4,5,6,7,8,9]). The relation of power output (P) and pedalling rate is parabolic and can be analysed by non-linear regression (ibid.). In cycling, these profiles illustrate the maximal resistance that an athlete can overcome at a certain cadence, and thus the maximal power output at that cadence.

In diagnostics, F/v and P/v profiles allow to describe important characteristics of performance, such as the theoretical maximal mean crank force ($F_{\max}$) and maximal pedalling rate (${\mathrm{PR}}_{\max}$) as y- and x-axis intercepts of the F/v function, the maximal mechanical power output ($P_{\max}$) as the apex of the P/v relation and the optimal pedalling rate (${\mathrm{PR}}_{\mathrm{opt}}$) as corresponding cadence [6].

Maximal force-velocity (F/v) and power-velocity (P/v) relations of cyclists are often established on a cycle ergometer in the laboratory utilizing a number (at least three to four efforts) of “fatigue-free” isovelocity maximal sprints at different cadences (usually ≤ 6 s, assuming a fatigue-free time interval of ≤ 6 s, with 5 min passive rest between efforts) [10,11,12,13,14,15,16,17]. This method allows a series of pedalling cycles to be recorded for each cadence tested. However, participants usually have to accelerate to the desired cadence by pedalling against the resistance of a large gear ratio and flywheel, and may reach the target cadence after only a few seconds, possibly in a fatigued state. This, combined with the relatively short rest between efforts, can lead to a fatigue-induced drop in performance that can affect the quality of the data [18].

Alternatively, these profiles can be determined using one single isoinertial sprint bout (usually ≤ 7 s) in which participants accelerate maximally from a standing or rolling position on a friction-loaded ergometer so that several movement velocities occur during the sprint [4,5,8,18,19]. Recent research has found slight improvement in the quality of F/v profiles for the isoinertial method when comparing the two methods, presumably due to the absence of multiple sprints after incomplete recovery [20]. Due to the similarity to the demands of acceleration from a standing or rolling position during competition, this acceleration method is considered highly suited for diagnostics in track cycling sprinting [8,19].

To determine track cycling sprint performance via maximal F/v and P/v profiles directly on the cycling track, Gardner and colleagues [8] transferred the acceleration method to the field and derived profiles from two maximal standing-start 65 m sprints on a cycling track conducted with a constant gear ratio of 48 (front)/14 (rear). They demonstrated high model quality and high agreement with profiles derived from laboratory testing using two 6 s-maximal isoinertial sprints on a cycle ergometer [8].

Following this approach, it seems possible to derive valid maximal F/v and P/v profiles from data collected during the first 7 s of a maximal (standing-start) sprint on the cycling track using pedalling rates ranging from 55–95 revolutions per minute (rpm) [8]. In accordance with the procedure suggested by Gardner and colleagues [8], a comparable method was used to determine the maximal F/v and P/v profiles in BMX cycling [9,16].

As was pointed out by a number of researchers, in order to obtain valid maximal F/v and P/v profiles performance should be considered under realistic conditions across the entire force-velocity spectrum, especially the two profile extremes (absolute strength and strength speed/speed) should be represented [2,18,21,22,23,24,25]. F/v profiles derived from data with maximal pedalling rates of 100 rpm or less may not accurately represent a cyclist’s potential at high to very high cadences.

Furthermore, the data used to determine maximal F/v profiles should be free of fatigue-related performance losses to validly depict maximal fatigue-free performance [18]. Our own research results show a deviation from the fatigue-free maximal F/v profile after ≤ 3 s of maximal exercise in highly trained sprint cycling athletes due to metabolic fatigue [26]. If data beyond 3 s are included in the regression analysis, this database is unlikely to represent the cadence-specific maximum in the cadence range reached after the onset of fatigue. This assumption is supported by the results of Kordi et al. [27], who calculated a significantly higher maximum cadence using the isovelocity method compared to the isoinertial method.

We assume that creating maximal F/v and P/v profiles in track cycling based on the data of a single maximal 65-m acceleration on the cycling track does not validly represent the fatigue-free maximal performance of an athlete and may over- or under-estimate important profile parameters such as maximal and optimal cadence due to two factors: (1) a limited cadence spectrum and (2) an excessive exercise duration represented in the data.

Collecting fatigue-free data across the entire force-velocity spectrum would require several short maximal sprint tests on the cycling track with significant different gears, analogous to the isovelocity method in the laboratory. Such a procedure would be less efficient in practice due to the time required and fatigue accumulated.

In the following, we present a possible integration of the most recent research findings into diagnostic procedures and aim to improve the validity of force-velocity profiles created for track sprint cyclists and to elucidate a potential independence of those profiles on the choice of gear ratio.

As a viable option for practical testing on the cycle track, we propose that the creation of maximal F/v profile can be based on a single maximal acceleration when additional fatigue-free data points at high cadences are included. Those data can exemplarily be derived from a high-cadence low-resistance sprint on a non-resisted (free) roller trainer (motoric test) during the daily warm-up program.

Furthermore, we test the impact of the gear ratio on the results and postulate that maximal F/v and P/v profiles can be produced with any gear ratio that an athlete can manage, as long as the minimum conditions of the approach are met.

## 2. Methods

### 2.1. Participants

Twelve elite track cycling sprinters (four females; 21.8 ± 1.3 yrs, body height: 1.75 ± 0.05 m, body weight: 73.1 ± 3.2 kg and eight males; age: 26.8 ± 3.6 yrs, body height: 1.83 ± 0.04 m, body weight: 88.4 ± 5.9 kg; crank length: 0.17 ± 0.00 m (means ± standard deviations)) took part in this investigation.

As our study design required high-level neuromuscular performance, only athletes who had demonstrated closely linear F/v profiles (R^2^ > 0.95) in previous tests in the laboratory and who had already competed in track cycling sprint events at international championships were included. All participants used their own bicycle, cycling shoes and pedals during the test.

The participants were requested to refrain from consuming alcohol and from intense training and asked to maintain their normal drinking and eating habits for 24 h prior to the experimental session. All participants provided their informed written consent to participate in this study, which was approved by the institute’s ethical committee and performed in accordance with the Declaration of Helsinki.

### 2.2. Exercise Protocol

After a standardized warm-up each subject completed a motoric test on a free roller and two series of three maximal 65-m sprints on an official cycling track (Frankfurt an der Oder, Germany).

### 2.3. Warm-Up

The warm-up prior to the tests involved 15 min of low-intensity cycling, with self-selected intensity within the recommended power ranges (1–1.5 W kg^−1^ bodyweight), followed by two 6-s maximal sprints on the track (starting from rolling at approximately 40 rpm). This warm-up and each series of sprints were separated by 10 min of cycling at low intensity and 10 min of passive recovery.

### 2.4. Motoric Test

After the warm-up, participants first performed two 6-s maximal sprints in a seated position on a free roller (separated by 5 min of passive recovery). The gear ratios used were chosen so that the athletes reached a cadence of ≥ 160 rpm within the first 3 s of the maximal sprint.

### 2.5. Track Sprints

Then, all athletes performed two series of three maximal 65-m sprints in a sitting position initiated from a rolling start at approximately 40 rpm at <100 W with their preferred leg first. Each sprint was interspersed by 5 min of passive recovery. All sprints were produced on the straight lines of the track. To investigate the validity of the profiles and elucidate the impact of gear selection, both sprint series were run with significantly different gear ratios. In the first series, a small gear (development of 6.76 ± 0.17 m, mean ± standard deviation) was selected for all athletes. In the second series, the athletes were asked to choose a high, but manageable gear for standing starts (development of 8.00 ± 0.48 m).

Throughout the testing, pedal force and crank velocity were measured continuously with a FES power meter. The system supplied by the Institute for Research and Development of Sports Equipment (FES) in Berlin captures the tangential force on the crank arm with a sample frequency of 200 Hz. From this high-resolution power-meter data, the mean tangential force F (N) at the left pedal, averaged per one revolution, as well as the corresponding mean pedal rate PR (rpm) was derived.

### 2.6. Data Processing

Two different methods, in the following referred to as models I and II, were used to derive maximal F/v and P/v profiles from the data: In model I following Gardner et al. [8], the F/v and P/v profiles were calculated based on the mean pedalling rate and the corresponding mean crank force during the first 6 s (in accordance to the traditionally assumed fatigue-free time interval) of each sprint on the track. In our proposed new method of model II, both the initial acceleration phase of each maximal sprint on the track and the motoric test were considered. From the track sprints, the first 3 or 4 cycles (≤ 3 s) with linear F/v relation were taken, and 1 or 2 fatigue-free cycles at pedal rates above 160 rpm from the motoric test were evaluated to establish the fatigue-free F/v and P/v profiles. As cadence is proportional to the tangential speed of motion velocity of the pedal, the profiles were based on the mean cadence PR and corresponding mean crank force F.

The force-velocity continuum was analysed in terms of linear (F/v profile) and non-linear (P/v profile) regression. The function

(1) $$F\left(v\right)=a\cdot \mathrm{PR}+b$$

approximates the fatigue-free relation of mean pedal force F and the movement velocity PR, where a < 0 reflects the decline in mean pedal force with increasing cadence and b the theoretical maximal mean pedal force.

Net power output P(v) was calculated by multiplying force F(v) times cadence PR:

(2) $$P\left(v\right)=a\cdot {\mathrm{PR}}^{2}+b\cdot \mathrm{PR}$$

The following equations were used to obtain various characteristics of performance for the best effort of each series: theoretical maximal mean pedal force $F_{\max}=F\left(0\right)=b$, theoretical maximal velocity of movement PR_max_ = −b·a^−1^, optimal cadence PR_opt_ = −b·(2a)^−1^ and maximal power output P_max_ = −b^2^(4a)^−1^. The best efforts were defined as those that indicated the highest peak performance. To evaluate the validity of the different approaches, the consistency of the profiles with raw data results was checked by P_max_ ≥ P_peak_.

### 2.7. Statistical Analyses

Values are reported as means ± standard deviations (SD). Normal distribution of the data was confirmed by Shapiro and Wilk testing and the Levene test for homoscedastity. Differences between the profiles created on the basis of the different runs and models were compared by one-way ANOVA for repeated measures with Bonferroni post-hoc analysis, with the subject’s sex as an interindividual factor; while the mean differences between these parameters were compared employing t-tests for dependent samples. Cronbach’s alpha and ICC were calculated to investigate the consistency of the profile parameters derived from series 1 and series 2. In all cases, the level of statistical significance was set at *p* < 0.05 and Cohen’s d (small = 0.2; medium = 0.5; large = 0.8) employed as a measure of effect size. The quality of the regression analyses was assessed by calculating the coefficient of determination R^2^. Mathematical analysis and statistical tests were processed using IBM SPSS statistics version 24 Software for Windows (SPSS Inc., Chicago, IL, USA) and Office Excel 2016 (Microsoft Corporation, Redmond, WA, USA).

## 3. Results

The gear ratios chosen for the first series of sprints of 54 ± 2 (front)/17 ± 1 (rear; development of 6.76 ± 0.17 m) resulted in a range of pedalling rate of 70 rpm to 150 rpm. In the second series conducted with a mean gear ratio of 56 ± 1(front)/15 ± 1(rear; 8.00 ± 0.48 m), the data points reflected pedalling rates between 50 rpm to 110 rpm. In the motoric test data points at pedalling rates between 165 rpm and 235 rpm were obtained.

Anthropometric data and the parameters of the fatigue-free F/v profile with corresponding model quality R^2^ calculated with model I and model II are presented in Table 1 for all athletes.

Although both models exhibited high-to-excellent linearity, statistically significant differences could be observed. Maximal force $F_{\max}$ (1507.51 ± 257.60 N and 1384.35 ± 276.84 N; *p* < 0.002; d = 2.555) and maximal net power P_max_ (1499.54 ± 373.17 W and 1623.84 ± 84; *p*<0.017, d = −1.711) were statistically significant higher and the slope of the function (a_I_ = −6.78 ± 1.17 and a_II_ = −5.24 ± 1.11; *p* < 0.003, d = −2.401) and PR_max_ (223.73 ± 27.11 rpm and 264.59 ± 23.17 rpm; *p* < 0.004; d = −2.427) statistically significant steeper with model I than model II. Both linear regressions produced high coefficients of determination, with R^2^ amounting to 0.93   0.06 for model I and to 1.00 for model II. Model II thus showed a higher, almost ideal explained variance (*p* < 0.003, d = −2.427).

Although fatigue-free maximal force (*p* < 0.002; d = 3.157), and maximal power output were higher in male athletes (*p* < 0.001; d = 4.235), no significant interindividual effects were observed for any of the parameters.

Deviations from the fatigue-free raw data at high cadences derived from the sprint with small gear ratio and the motoric test were smaller for model II. In the case of a fatigue-free mean pedal force of 250.34 ± 84.87 N at a cadence of 215.65 ± 18.95 rpm derived from the motoric test, these deviations were 224.07 ± 163.89 vs. 1.39 ± 1.33 N, respectively (*p* < 0.002, d = 2.997). Considering a fatigue-free high cadence data point derived from smaller geared sprint at a mean cadence of 141.00 ± 6.71 rpm and corresponding mean pedal force of 667.91 ± 126.88 N, these deviations were 106.35 ± 83.16 vs. 9.41 ± 10.06 N, respectively (*p* < 0.002, d = 2.312).

**Figure 1:**
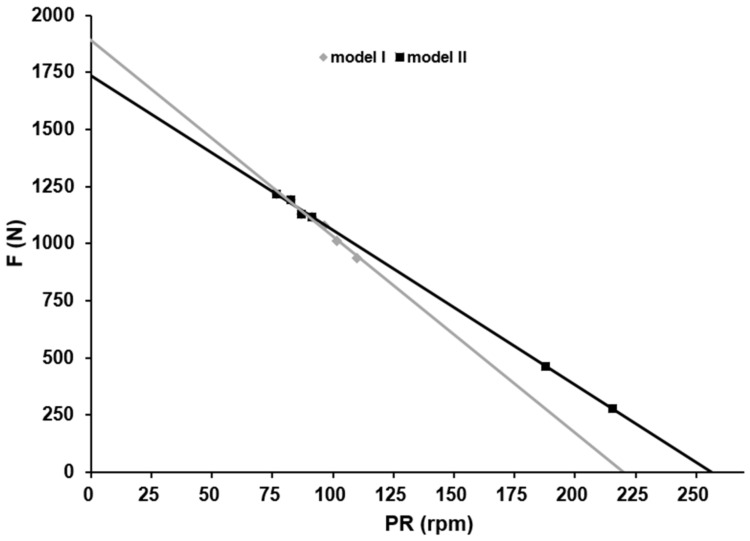
Comparison of the F/v profiles calculated for athlete A with model I (grey line) or model II (black line) reveals that the former overestimates and underestimates force development at slow and fast pedalling rates, respectively. The values obtained with model I differs from the measured mean pedal force of 176 N at 232 rpm by more than 40%, whereas the corresponding values obtained with model II shows a deviation of <2%.

A representative example of these differences is depicted in Figure 1. For all athletes, the F/v profile was significantly steeper and the maximal pedalling rate significantly lower with model I than with model II.

**Figure 2:**
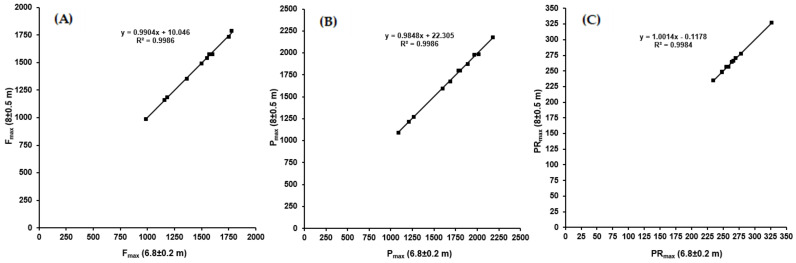
(**A**–**C**) The results of linear regression analysis for the main profile parameters of the profiles derived from the best sprint and calculated with model II for the different series driven with different gear ratios. (**A**) Theoretical maximal mean pedal force F_max_; (**B**) maximal power output P_max_ and (**C**) theoretical maximal pedalling rate PR_max_.

Comparing the F/v and P/v profiles for the best sprint in each series no significant systematic difference in the characteristic values was observed (*p* > 0.154). The results of linear regression analysis for the main profile parameters are shown in Figure 2.

Cronbach’s alpha and intraclass correlation coefficients (ICC) were calculated as 1.00 for parameter a and b, which indicate excellent model consistency. The parameters of the F/v profile derived from the best sprint of each series calculated with model II are shown in Table 2.

## 4. Discussion

In agreement with Gardener and colleagues [8], both of our models I and II demonstrated high- to-excellent linearity, but the values obtained with the adjusted model II were statistically higher. In particular, the higher level of maximal force with a steeper slope of the F/v profile based on model I resulted in mean pedal forces at high cadences that were lower than those the athletes actually realized in the motoric test. With model I the risk of underestimating an athlete’s level of performance at high cadences is high and may result in an incorrect fatigue-free maximal cadence. As an elite track sprinter’s maximal and optimal cadences (${\mathrm{PR}}_{\mathrm{opt}}=0.5\cdot {\mathrm{PR}}_{\max}$) are sensitive indicators of performance for both training and competition [28], such inaccuracy may lead to substantial errors, for example, when selecting a non-optimal gear ratio [29].

When creating F/v profiles for sprint cyclists, it appears necessary to include at least one data point at a realistically high pedalling rate associated with speed strength or speed [18,22,23,24]). Model I, basing on cadences of 40–150 rpm, reflects performance in the range maximal strength or power, obtaining performance at cadences < 160 rpm only by extrapolation. Since the patterns of muscular recruitment differ at different velocities [30], this can lead to an inaccurate representation of performance, particularly in the extrapolated portion of the F/v profile.

Moreover, model I involves data from the first 6 s of a maximal sprint on a cycling track. Although Seck et al. [4], Gardner et al. [8] and Debraux et al. [9] reported a linear developement of the force-velocity data within those 6 to 7 s, which is also confirmed by our results, we recently showed that professional track cycling sprinters leave their fatigue-free F/v profile after a maximum of 3 s of maximal cycling due to a reduced energy supply [26]. The high linearity of the F/v profile derived from a 6 to 7 s maximal sprint suggests validity even though an athlete did not perform maximally for several seconds due to fatigue.

Both problems associated with model I can lead to an inaccurate F/v profile as shown in Figure 1. The findings with model II suggest incorporating high fatigue-free cadences (>160 rpm), so the risk for such error is significantly reduced. Using data gathered during the first 6 s together with data collected at a very high crank velocity (i.e., a combination of models I and II) reduces the risk of obtaining an inaccurate maximal cadence (model I: 222 rpm, model II: 264 rpm, combined model: 263 rpm). This finding emphasizes the importance of including data from high pedalling rates in order to obtain a valid estimate of the optimal pedalling rate.

Due to the differences between the models, the mean extrapolated maximal cadences found in a sitting position of 264 rpm exceeds the values previously reported for field measurements of 256 rpm or 244 rpm, respectively, for sprinting in a standing position [8,9]. Our own research indicates even higher maximal cadences in standing than in sitting position (unpublished data), underlining the possibility of an underestimation of the actual maximal cadence with the previous approaches. The smaller R^2^ values reported by Gardner et al. [8] and Debraux et al. [9] (R^2^ < 0.984) support the possibility of a fatigue bias present in the data used to generate F/v and P/v profiles.

Dorel and colleagues [6] used 5 s maximal sprint and thereby probably not only fatigue-free data to create maximal F/v and P/v profiles. Their results for maximal mean pedal force, maximal power and maximal pedalling rate compare well with our findings. The high cadence range reflected in their data (<50 to >200 rpm) derived from three maximal sprints against different forces applied to a friction belt seems to reduce or prevent calculation errors.

When creating valid maximal F/v profiles, it seems highly important to represent the different muscular recruitment patterns by data points in different cadence ranges.

Despite the comparatively good prediction of the performance at pedalling rates >160 rpm by model II and the high model validity suggested by it overall, for one athlete the use of data points at very high cadences led to a reduction in the F/v profile slope. This may indicate a potential neuromuscular and/or coordinative deficit of the athlete, hindering utilization the actual strength speed and speed performance potential. This is an example of the versatility of F/v profiles, which may be used to identify such potentials.

Comparing the best profiles for the two individual series of sprints reveals an independence of the choice of gear ratio. The statistical analysis of the profiles indicates high consistency.

In a maximal cycling effort, the gear ratio determines the resistance, which must be overcome at any given pedalling rate. Maximal cycling with different gear ratios leading to pedalling at different rates with different corresponding pedal forces seems to be analogous to the choice of different loads in strength exercises. If the range of cadences is sufficiently high to represent different neuromuscular recruitment patterns, the profile derived should be independent of the gear ratio. Transferring the assignment of movement velocity ranges to strength abilities as suggested by Mann [23] using the mean F/v profile with a maximal pedalling rate of 265 rpm, the low-frequency cadence range up to 80 rpm appears to be determined by strength, the cadence range from 80 to 160 rpm by power, cadences of between 160 to 220 rpm by speed strength and cadences > 220 rpm by speed.

The database used in model II represents the entire force-velocity spectrum and the very high model quality supports its validity. We deem our approach suitable for creating baseline profiles that allow the assessment of fatigue-free performance during sprinting on a cycling track.

## 5. Limitations

At present, the application of our model is limited to maximal sprints on a cycling track and has only been validated for a relatively small group of elite athletes. Further validation of the reliability of our approach, as well as examination of its potential applicability to other conditions and sports is required.

Due to the measuring system used, the profiles were derived from the left crank only, possibly measuring combined propulsive forces of both legs. This may reduce comparability to profiles derived from both legs independently. It may further reduce comparability to profiles derived from strength exercise, where designated muscles or muscle chains are analyzed.

The determination of maximal F/v and P/v profiles requires athletes to perform with maximal neuromuscular control. If an athlete’s performance is sub-maximal due to, e.g., deficits in neuromuscular control, a valid representation of maximal performance is not possible. This is reflected in a comparatively poor model quality. In such cases, the need for regeneration (if the deficit is due to fatigue) or training interventions to improve neuromuscular control (if the deficit is a performance reserve) can be deduced from the diagnostic results.

The fatigue-free F/v profile of an athlete is not constant but depends on the current functional state of the nervous system (muscle fibre recruitment, intermuscular coordination, synchronisation, co-contractions) as well as tendons and the muscle system itself (muscle contraction mechanics, energy flow in the muscle cell). Central and peripheral fatigue, hormonal status (stress level, time of day), training conditions and the potential cross-over effects of different exercises can influence the values of individual indicators of maximal performance, including cadence, indicating the need for frequent measurements.

## 6. Practical Applications

In track cycling, maximal F/v and P/v profiles allow the description of important performance characteristics. When creating these profiles, a sufficiently large cadence range (mean pedal forces with associated cadence representing maximum strength to speed strength or speed should be included as data points) and reflecting an athlete’s maximal, i.e., fatigue-free, performance should be ensured.

As a viable option for practical testing within the available time budget, we suggest deriving maximal F/v and P/v profiles based on a single maximal sprint with any gear an athlete can manage combined with a low resistance, high cadence motoric test, ensuring the athletes’ performance in a fatigue-free state at pedalling rates above 160 rpm. Our practical experience shows that such a motoric test can be integrated as part of the warm-up routine.

## 7. Conclusions

In track cycling, F/v and P/v profiles provide valuable insight into performance characteristics. The procedure for the creation of such profiles, referred to as model II in this study, is accurate, independent of the gear ratio and suited for the creation of baseline profiles for assessing fatigue-free performance on the cycling track. Creating F/v profiles based on the first 3 to 4 crank revolutions (≤3 s) of a single short maximal sprint on the track with an arbitrary gear ratio the athlete is capable to manage, combined with low-resistance high-cadence test, ensures a sufficiently wide cadence range and protects against fatigue-related data bias.

## Figures and Tables

**Table 1:** Anthropometric data and parameters of the F/v profiles derived from the best effort of series 2 calculated with model I and model II for each participant. The best efforts were defined as those with highest calculated maximal power output.

				Model I			Model II		
Part.	Age (yrs)	Height (cm)	Bodyweight (kg)	a (N rpm^−1^)	b (N)	R^2^	a (N rpm^−1^)	b (N)	R^2^
1	29	186	92	−8.52	1750	0.88	−6.16	1578	1.00
2	29	178	81.6	−5.75	1406	0.98	−5.12	1353	1.00
3	25	187	95	−8.14	1852	0.97	−6.77	1736	1.00
4	33	182	91.8	−6.10	1710	0.92	−7.19	1788	1.00
5	22	189	89	−6.43	1561	0.95	−5.50	1491	1.00
6	25	179	84.7	−6.98	1516	0.84	−4.88	1355	1.00
7	29	184	95	−8.95	1863	0.82	−4.70	1539	1.00
8	22	182	78	−6.74	1637	0.94	−5.91	1577	1.00
9	21	177	74	−5.37	1252	0.88	−4.77	1184	1.00
10	23	167	68	−5.00	1036	0.99	−3.10	862	1.00
11	23	176	77	−6.70	1302	0.98	−4.95	1163	1.00
12	20	180	73.2	−6.66	1206	1.00	−3.84	985	1.00

Abbreviations: Part.: participants, a: slope of the F/v profile, b: intercept of the y-axis of the F/v profile, R^2^: coefficient of determination.

**Table 2:** Parameters of the F/v profiles derived from the best efforts of series 1 and series 2 calculated with model II for each subject. Best efforts were defined as those with highest calculated maximal power output.

	Series 1				Series 2			
Part.	a (N rpm^−1^)	b (N)	R^2^	Developm. (m)	a (N rpm^−1^)	b (N)	R^2^	Developm. (m)
1	−6.16	1571.07	1.00	7.0	−6.16	1578.39	1.00	8.4
2	−5.17	1361.52	1.00	6.8	−5.12	1352.67	1.00	8.4
3	−6.77	1750.52	1.00	6.8	−6.77	1736.32	1.00	8.7
4	−7.16	1777.11	1.00	7.0	−7.19	1787.65	1.00	8.4
5	−5.56	1500.26	1.00	6.7	−5.50	1490.74	1.00	7.1
6	−4.89	1361.38	1.00	6.7	−4.88	1355.12	1.00	8.4
7	−4.74	1546.91	1.00	6.7	−4.70	1539.41	1.00	7.6
8	−6.01	1598.01	1.00	6.5	−5.91	1577.43	1.00	8.0
9	−4.77	1183.16	1.00	6.7	−4.77	1183.78	1.00	7.5
11	−4.94	1157.78	1.00	7.0	−4.95	1163.14	1.00	7.7
12	−3.83	983.24	1.00	6.5	−3.84	985.18	1.00	7.8

Abbreviations: Part.: participants, a: slope of the F/v profile, b: intercept of the y-axis of the F/v profile, R^2^: coefficient of determination. One subject had to be excluded because of failed measurements in the first series.

## Data Availability

The datasets generated and/or analysed during the current study can be obtained from the corresponding author upon reasonable request.

## Abbreviations

a	Slope of the F/v profile
F/v	Force-velocity
F	Force
$F_{\max}$	Theoretical maximal pedal force
P	Power output
$P_{\max}$	Maximal power output
PR	Pedalling rate; cadence
${\mathrm{PR}}_{\max}$	Maximal pedalling rate; maximal cadence
${\mathrm{PR}}_{\mathrm{opt}}$	Optimal pedalling rate; optimal cadence
P/v	Power-velocity
v	Velocity
rpm	Revolutions per minute
